# Supplementary material for: Acute respiratory distress syndrome readmissions: A nationwide cross-sectional analysis of epidemiology and costs of care
Source: PLoS One. 2022 Jan 25;17(1):e0263000. doi: 10.1371/journal.pone.0263000 (PMC8789165; doi:10.1371/journal.pone.0263000)
Supplement: S1 Table — (DOCX) [file pone.0263000.s001.docx]

| **S1 Table. Demographics on all NRD records vs case definitions.** | | | | |
| --- | --- | --- | --- | --- |
| **Factor** | **N, Total** | **All others in NRD (N =35,633,848)** | **Cases (N = 27,058)** | **p-value** |
| Sex, % (95% CI) | 35,660,906 |  |  | ***<0.0001^c^*** |
| Male | 15,435,939 | 43.3 (42.9, 43.6) | 52.4 (51.5, 53.4) |  |
| Female | 20,224,967 | 56.7 (56.4, 57.1) | 47.6 (46.6, 48.5) |  |
| Died during hospitalization, % (95% CI)* | 35,643,858 |  |  | ***<0.0001^c^*** |
| No | 34,951,792 | 98.1 (98.0, 98.1) | 62.3 (61.0, 63.6) |  |
| Yes | 692,066 | 1.9 (1.9, 2.0) | 37.7 (36.4, 39.0) |  |
| Disposition of patient (uniform), % (95% CI)* | 35,643,858 |  |  | ***<0.0001^c^*** |
| Discharged to home or self care | 25,191,902 | 70.7 (70.0, 71.4) | 21.7 (20.6, 22.9) |  |
| Transfer: short-term hospital | 285,434 | 0.80 (0.76, 0.84) | 3.5 (3.0, 4.0) |  |
| Transfer: other type of facility | 4,506,502 | 12.6 (12.3, 13.0) | 24.0 (23.0, 24.9) |  |
| Home health care | 4,516,368 | 12.7 (12.2, 13.1) | 12.0 (11.3, 12.6) |  |
| Against medical advice | 445,609 | 1.3 (1.2, 1.3) | 1.03 (0.85, 1.2) |  |
| Died in hospital | 692,066 | 1.9 (1.9, 2.0) | 37.7 (36.4, 39.0) |  |
| Discharged alive, destination unknown | 5,976 | 0.02 (0.01, 0.03) | 0.10 (0.03, 0.16) |  |
| Primary expected payer (uniform), % (95% CI)* | 35,615,445 |  |  | ***<0.0001^c^*** |
| Medicare | 14,442,804 | 40.5 (39.6, 41.5) | 49.5 (48.2, 50.8) |  |
| Medicaid | 8,076,916 | 22.7 (21.9, 23.5) | 19.7 (18.6, 20.7) |  |
| Prvt. Ins/HMO | 10,624,496 | 29.8 (29.0, 30.7) | 23.4 (22.3, 24.6) |  |
| Self-pay | 1,249,027 | 3.5 (3.3, 3.7) | 3.8 (3.3, 4.3) |  |
| No Charge | 143,843 | 0.40 (0.30, 0.51) | 0.43 (0.26, 0.60) |  |
| Other | 1,078,360 | 3.0 (2.7, 3.3) | 3.2 (2.7, 3.6) |  |
|  |  |  |  |  |
| Patient Location: NCHS Urban-Rural Code, % (95% CI) | 35,555,726 |  |  | ***0.0022^c^*** |
| Large Central Metro | 9,013,000 | 25.3 (23.3, 27.4) | 25.8 (23.6, 28.1) |  |
| Large Fringe Metro | 9,296,811 | 26.1 (24.0, 28.3) | 24.6 (21.9, 27.2) |  |
| Medium Metro | 7,757,206 | 21.8 (19.8, 23.8) | 20.6 (18.4, 22.7) |  |
| Small Metro | 3,612,788 | 10.2 (8.9, 11.4) | 9.8 (8.6, 11.0) |  |
| Micropolitan | 3,226,199 | 9.1 (8.5, 9.6) | 10.5 (9.4, 11.6) |  |
| Noncore | 2,649,721 | 7.5 (6.9, 8.0) | 8.7 (7.6, 9.8) |  |
| Elective versus non-elective admission, % (95% CI)* | 35606277 |  |  | ***<0.0001^c^*** |
| No | 28,220,049 | 79.2 (78.5, 80.0) | 91.9 (91.1, 92.6) |  |
| Yes | 7,386,228 | 20.8 (20.0, 21.5) | 8.1 (7.4, 8.9) |  |
| Median household income national quartile for patient ZIP Code, % (95% CI)* | 35,199,768 |  |  | ***0.0108^c^*** |
| First quartile | 10,599,244 | 30.1 (28.7, 31.6) | 32.1 (30.1, 34.0) |  |
| Second quartile | 9,196,286 | 26.1 (25.2, 27.0) | 26.2 (24.6, 27.7) |  |
| Third quartile | 8,674,080 | 24.6 (23.8, 25.5) | 23.7 (22.5, 25.0) |  |
| Fourth quartile | 6,730,158 | 19.1 (17.8, 20.5) | 18.0 (16.3, 19.7) |  |
| Case Definition: Any ARDS diagnosis and Age Eligible (>17y) having any codes for mechanical ventilation and/or endotracheal intubation. Totals presented are weighted unless otherwise specified. NCHS Urban-Rural definitions are based on 2013 classifications found at <https://www.cdc.gov/nchs/data_access/urban_rural.htm>  *Data not available for all subjects. Unweighted frequencies: Died during hospitalization = 11,686; Disposition of patient (uniform) = 11,686; Primary expected payer (uniform) = 23,478; Patient Location: NCHS Urban-Rural Code = 62,141; Elective versus non-elective admission = 19,440; Median household income national quartile for patient ZIP Code = 246,279. P-values: a=linear regression; b=linear regression with log transformation; c=Rao-Scott chi-square test. | | | | |
