# Supplementary material for: Acute respiratory distress syndrome readmissions: A nationwide cross-sectional analysis of epidemiology and costs of care
Source: PLoS One. 2022 Jan 25;17(1):e0263000. doi: 10.1371/journal.pone.0263000 (PMC8789165; doi:10.1371/journal.pone.0263000)
Supplement: S2 Table — (DOCX) [file pone.0263000.s002.docx]

| **S2 Table: Age-stratified mortality of records meeting case definition during index admission and readmission** | | | |
| --- | --- | --- | --- |
| **Factor** | **In-Hospital mortality N; % (95% CI)** | **Readmission mortality N; % (95% CI)** |  |
| **Overall** | 9,439; 37.5% (36.2, 38.8) | 244.0; 8.45% (6.7, 10.0) |  |
| **Age Groups** |  |  |  |
| **18-34 years** | 616.7; 24.1% (21.4, 26.8) | SUPPRESSED |  |
| **35-44 years** | 587.2; 24.7% (21.4, 27.9) | SUPPRESSED |  |
| **45-54 years** | 1,277; 30.5% (28.2, 32.8) | 36.4; 7.0% (3.8, 10.1) |  |
| **55-64 years** | 2,266; 39.6% (37.4, 41.6) | 50.9; 7.3% (1.4, 4.6) |  |
| **65-74 years** | 2,267; 44.3% (41.7, 46.8) | 58.1; 9.6% (5.9, 13.3) |  |
| **75 and older** | 2,426; 46.5% (44.3, 48.7) | 87.1; 16.6% (11.9, 21.3) |  |
| *Data not available for all subjects. Frequencies and percentages presented are weighted counts. Rows with cells having 10 or less in frequency are suppressed per HCUP requirements | | |  |
