# Supplementary material for: Acute respiratory distress syndrome readmissions: A nationwide cross-sectional analysis of epidemiology and costs of care
Source: PLoS One. 2022 Jan 25;17(1):e0263000. doi: 10.1371/journal.pone.0263000 (PMC8789165; doi:10.1371/journal.pone.0263000)
Supplement: S3 Table — (DOCX) [file pone.0263000.s003.docx]

**S3 Table. Disposition of Index Admission: Bivariate association of disposition and readmission**

| **Factor** | **No readmissions** | **Readmissions** |  |
| --- | --- | --- | --- |
| Discharged alive to home, self care or unknown* | 36.5% | 30.7% | Overall p=0.0010 |
| Transfer: Short-term hospital | 5.3% | 5.2% |  |
| Transfer: other type of facility | 37.6% | 41.1% |  |
| Home health care | 19.0% | 20.8% |  |
| Against medical advice | 1.6% | 2.2% |  |

*Combined category of those discharged to home or self care and those discharged alive but whereabouts unknown. This was done to eliminate zero counts in the latter. Rao-Scott chi-square was used for the overall measure of association. Percents were rounded to achieve 100% per column.
