# Supplementary material for: Acute respiratory distress syndrome readmissions: A nationwide cross-sectional analysis of epidemiology and costs of care
Source: PLoS One. 2022 Jan 25;17(1):e0263000. doi: 10.1371/journal.pone.0263000 (PMC8789165; doi:10.1371/journal.pone.0263000)
Supplement: S4 Table — (DOCX) [file pone.0263000.s004.docx]

**S4 Table. Bivariate association of APR-DRG of the index admission and 30-day readmission**

| **Factor**  **(APR-DRG)** | **% of Index Admissions** | **% Index Admissions w/o 30-d readmission** | **% Index Admissions w/**  **30-d readmission** | **p-value, association with readmission** |
| --- | --- | --- | --- | --- |
| *720: Septicemia & disseminated infections* | 17.7%  (16.6, 18.8) | 18.3%  (17.1, 19.5) | 15.1%  (13.2, 17.1) | 0.0042** |
| *137: Major respiratory infections & inflammations* | 1.23%  (0.97, 1.49) | 1.31%  (1.01, 1.62) | 0.84%  (0.42, 1.26) | 0.11 (NS) |
| *139: Other pneumonia* | 2.6%  (2.3, 3.0) | 2.6%  (2.2, 3.0) | 2.8%  (2.0, 3.6) | 0.74 (NS) |
| *190: Acute myocardial infarction* | 1.07%  (0.84, 1.30) | 0.98%  (0.75, 1.22) | 1.43%  (0.85, 2.02) | 0.11 (NS) |
| *174: Perc. coronary intervention w AMI* | SUPPRESSED | SUPPRESSED | SUPPRESSED | SUPPRESSED |
| *282: Disorders of pancreas except malignancy* | SUPPRESSED | SUPPRESSED | SUPPRESSED | SUPPRESSED |
| *469: Acute kidney injury* | 0.62%  (0.43, 0.80) | 0.52%  (0.33, 0.70) | 1.06%  (0.46, 1.65) | 0.0335 (NS) |

Factors: All results are presented as percentage with factor present and 95% confidence interval. Rows with cells having 10 or less in frequency are suppressed per HCUP requirements. **Statistically significant association of factor and readmission using Rao-Scott chi-square. (NS) Not statistically significant p<0.01 applying Bonferroni correction for multiple comparisons.
