# Supplementary material for: Acute respiratory distress syndrome readmissions: A nationwide cross-sectional analysis of epidemiology and costs of care
Source: PLoS One. 2022 Jan 25;17(1):e0263000. doi: 10.1371/journal.pone.0263000 (PMC8789165; doi:10.1371/journal.pone.0263000)
Supplement: S5 Table — All results are weighted. Total costs are unadjusted. (DOCX) [file pone.0263000.s005.docx]

**S5 Table. Index admission total cost and length of stay (LOS) differences across age groups for those with a readmission. All results are weighted. Total costs are unadjusted.**

| **Age Group**  **(level)** | **N** | **Mean Total Cost** | **Mean Total Cost 95% C.I. ($)** | **Mean LOS, (days)** | **Mean LOS**  **95% C.I.** |
| --- | --- | --- | --- | --- | --- |
| **Age 18 – 34y^1^** | 260.9 | $86,475^4-6^ | (80,964, 91,986) | 21.89^5,6^ | (20.67, 23.15) |
| **Age 35 – 44y^2^** | 260.7 | $86,385^4-6^ | (80,139, 92,630) | 23.04^5,6^ | (21.87, 24.21) |
| **Age 45 – 54y^3^** | 515.6 | $80,234^5,6^ | (76,305, 84,162) | 22.36^5,6^ | (21.55, 23.18) |
| **Age 55 – 64y^4^** | 687.6 | $76,204^1,2,5,6^ | (72,263, 80,145) | 21.92^5,6^ | (20.78, 23.05) |
| **Age 65 – 74y^5^** | 581.8 | $61,956^1-4,6^ | (59,141, 64,770) | 18.02^1-4,6^ | (17.31, 18.72) |
| **>Age 75y^6^** | 492.8 | $42,813^1-5^ | (40,830, 44,795) | 14.08^1-5^ | (13.52, 14.65) |

Superscripts for Mean Total Cost reflect significantly different comparisons (all p≤0.005) in levels of mean total cost using weighted regression models on age groups. Comparisons reflect differences in least squares means of total cost between age group levels.

Superscripts for Mean LOS reflect significantly different comparisons (p<0.0001) in levels of mean LOS using weighted regression models on age groups. Comparisons reflect differences in least squares means of LOS between age group levels.
