# Supplementary material for: Acute respiratory distress syndrome readmissions: A nationwide cross-sectional analysis of epidemiology and costs of care
Source: PLoS One. 2022 Jan 25;17(1):e0263000. doi: 10.1371/journal.pone.0263000 (PMC8789165; doi:10.1371/journal.pone.0263000)
Supplement: S6 Table — (DOCX) [file pone.0263000.s006.docx]

**S6 Table. Readmission total cost regression modeling estimates**

| **Factor** | **Impact ($)** | **Factor** | **Impact ($)** |
| --- | --- | --- | --- |
| Baseline cost | $12,413 | Male | $4,590 |
| LOS per day | $130 | Female | $0 |
| Early Readmission | $16,919 |  |  |
| **Age and Mortality combined (choose one)** |  | **Insurance (choose one)** |  |
| Age 18-44 and survived | $5,454 | -Medicare | -$4415 |
| Age 18-44 and died | $62,860 | -Medicaid | -$3481 |
| Age 45-54 and survived | $2,251 | -Private Insurance^#^ | $3593 |
| Age 45-54 and died | $14,596 | -Self-pay | -$19852 |
| Age 55-64 and survived | $4,055 | -No Charge | -$23551 |
| Age 55-64 and died | $19,929 | -Other | $0 |
| Age 65-74 and survived | $5,865 | **Location (choose one)** |  |
| Age 65-74 and died | $21,435 | -Large Central Metro | $11,720 |
| Age 75+ and survived | $0 | -Large Fringe Mero | $10,894 |
| Age 75+ and died | $5,123 | -Medium Metro | $7,298 |
|  |  | -Small Metro | $3,329 |
|  |  | -Micropolitan | $1,688 |
|  |  | -Noncore | $0 |
| **Comorbidity at re-admission (choose all that apply)** | | | |
| Congestive Heart Failure^#^ | $733 | Metastatic cancer | -$4,625 |
| Valvular disease | $4,755 | Solid tumor w/o metastasis^#^ | -$5,336 |
| Pulmonary circulation disease | $3,244 | Rheumatoid arthritis/ collagen vas. | -$1872 |
| Peripheral vascular disease^#^ | -$553 | Coagulopathy | $9,347 |
| Paralysis | -$1,539 | Obesity | $4,847 |
| Other neurological disorders | -$6,707 | Weight loss^#^ | -$648 |
| Chronic pulmonary disease | -$4,206 | Fluid/electrolyte disord. | $4,713 |
| Diabetes w/o chronic complications^#^ | $327 | Chronic blood loss anemias | $15,003 |
| Diabetes w/chronic complic. | -$3,999 | Deficiency anemias | -$1,570 |
| Renal failure | -$5,360 | Alcohol abuse | -$2,808 |
| Liver disease | -$4,144 | Drug abuse | -$9,505 |
| Peptic ulcer disease excl. bleed | $4,637 | Psychoses | -$7,200 |
| AIDS | -$11,245 | Depression | -$1,651 |
| Lymphoma | $10,540 | Hypertension | -$3,883 |
| #not statistically significant. All other terms were significant p<0.05. Linear regression for readmission total charge was adjusted for the survey design. | | | |

**Supplementary Analysis: Regression Model for Readmission Costs**

***Readmission Total Cost*** *= intercept + LOS + Female + PublicIns. + NCHS_Location + AgeGroup(5 levels) + Early Readmission + Readmit_mortality + Elixhauser comorbidities (listed below) + (Interaction: AgeGroup(5 levels) * Readmit_mortality)*

Due to missing data, 1,551 records (2785.5 weighted readmissions, a 3.6% loss of representation) were used in the model. Nearly all model terms, including the interaction of Age and readmission mortality, were statistically significant but estimates were not unique (i.e. other values could be calculated as a linear combination of terms.) A baseline patient reflects a female, age 75 years and over, readmitted between day 3 and 30 after discharge from the index admission, having non-public/non-private (other) insurance, non-core or rural residence, without comorbidities, surviving readmission and discharged home.
