# Supplementary material for: Acute respiratory distress syndrome readmissions: A nationwide cross-sectional analysis of epidemiology and costs of care
Source: PLoS One. 2022 Jan 25;17(1):e0263000. doi: 10.1371/journal.pone.0263000 (PMC8789165; doi:10.1371/journal.pone.0263000)
Supplement: S7 Table — (DOCX) [file pone.0263000.s007.docx]

**S7 Table. Example charge calculation.**

| **Factor** | **Impact ($)** |
| --- | --- |
| Baseline charge | $12,413 |
| LOS per day * 7 days | $908 |
| Early Readmission (yes) | $16,919 |
| Female | $0 |
| Age 18-44 and survived | $5,454 |
| Medicaid | -$3,481 |
| Large Central Metro | $11,720 |
| Obesity | $4,847 |
| Fluid/electrolyte disord. | $4,713 |
| Hypertension | -$3,883 |
| **TOTAL** | $49,610 |

For example, a post-partum female age 18-44 with Medicaid, readmitted to the same large, central metropolitan hospital within two days after discharge (early readmission) with active comorbidities of hypertension, fluid & electrolyte disorder, and obesity, with a stay of 7 days (LOS) and discharged home (survived) would have had an estimated total charge of $49,610 as shown in Table S7. Had she not been an early admit, the estimated total charge would have been $32,691.
