# Supplementary figures and images for: Acute respiratory distress syndrome readmissions: A nationwide cross-sectional analysis of epidemiology and costs of care
Source: PLoS One. 2022 Jan 25;17(1):e0263000. doi: 10.1371/journal.pone.0263000 (PMC8789165; doi:10.1371/journal.pone.0263000)

**S1 Figure. Age and Mortality Coefficient for Readmission Cost Regression Model**


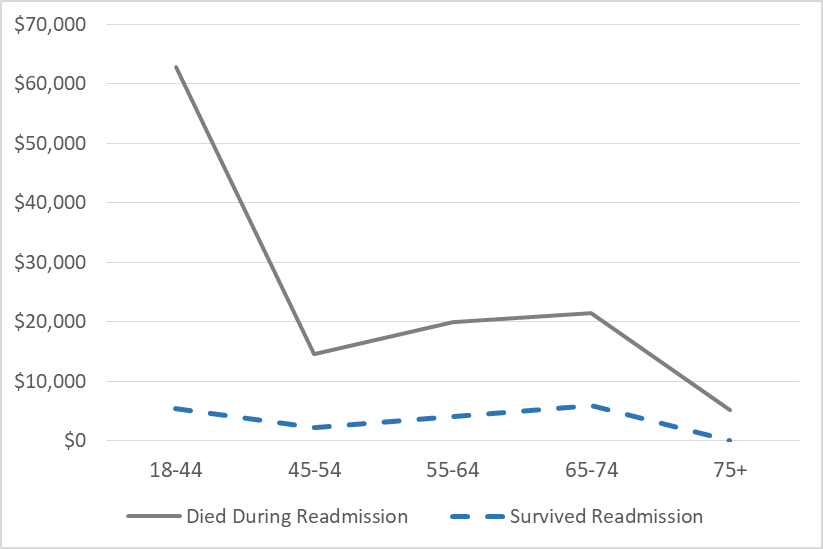

Supplement: S1 Fig — (DOCX) [file pone.0263000.s008.docx]
