# Supplementary material for: Acute respiratory distress syndrome readmissions: A nationwide cross-sectional analysis of epidemiology and costs of care
Source: PLoS One. 2022 Jan 25;17(1):e0263000. doi: 10.1371/journal.pone.0263000 (PMC8789165; doi:10.1371/journal.pone.0263000)
Supplement: Attachment — Submitted filename: PLOS review.docx [file pone.0263000.s009.docx]

In this paper, Dr Siuba and colleagues have used the HCUP’s NRD, which contains discharge abstract data for over 36 million weighted discharges, to describe the rate of 30-day readmission after an admission to hospital with a diagnosis of ARDS (defined using ICD-10 codes and procedure codes for intubation or tracheostomy). They also sought to explore the costs associated with both the admission to hospital for ARDS and a subsequent readmission. They identified 25,170 index episodes, with 9,439 dying during index admission, and 2889 experiencing a readmission within 30 days. 14.3% of the readmissions occurred within the first two days following discharge, and most had codes for the same most responsible diagnosis as was noted for the index admission. They identified some predictors of readmission using a multivariable logistic regression model that notably demonstrated that older age, and longer stay in hospital were associated with higher hospital costs. They found that the mean cost of hospitalization for ARDS was $71,004, and the mean cost of readmission was 26,971. Costs stratified by age demonstrated that younger groups had higher costs as well as longer mean length of stays than older groups. They also conducted a regression analysis to predict readmission costs based on various covariates.

The study question is important, and well-justified.

Major comments:

1. I would suggest that the authors rearrange the methods section slightly so that it follows more closely the EQUATOR checklist. I don’t see any descriptions of considerations of bias, or confounding, and these should be addressed.
2. The authors have identified some interesting trends, particularly that costs were higher among younger patients, who also had longer lengths of stay. Do the authors have any thoughts on how age and length of stay might interact in the causal pathway to costs?
3. Does the database only provide the total cost for an admission, or are the costs broken down into categories of costs? If they can be broken down, I would suggest describing the breakdown for the total cohort, as well as for the group that die during index admission, discharged with no readmission and those with a readmission. You might want to also describe costs for readmission stratified by early versus later readmission.
4. A significant limitation of this study is that health care utilization costs and readmission are likely predicted by previous healthcare use. Previous research has demonstrated that high-users of healthcare, and those who have higher health care costs (High cost users) are likely to remain high-cost users. Can they do a look back and identify previous healthcare utilization and costs for their cohort, and then either stratifiy or control for this in their models? Previous work (eg: <https://www.cmaj.ca/content/188/3/182>) has demonstrated that of 45% of high-cost users remain high-cost users over the next two years.

- The authors have described total costs, but have not described the attributable costs of ARDS. These costs are heavily confounded by the costs associated with underlying medical comorbidities, previous healthcare spending, hospitalization in general, and are not described relative to the costs associated for other non-ARDS types of hospitalizations. They might want to consider a matched analysis comparing costs of hospitalization for ARDS to a group hospitalized for other causes. (See this as an example of costing study employing this methodology:  [10.1097/CCM.0000000000004777](https://doi.org/10.1097/ccm.0000000000004777))

Minor:

1. Is it possible that patients were readmitted to a hospital that might not have reported discharge abstract data to this data set, and thus had a missed readmission? Or does the dataset include all data from all hospitals?
2. There is no mention of the cost findings in either the take-home section or the abstract.
